# PPARα activator irbesartan suppresses the proliferation of endometrial carcinoma cells via SREBP1 and ARID1A

**DOI:** 10.32604/or.2023.026067

**Published:** 2023-05-24

**Authors:** YU LU, TSUTOMU MIYAMOTO, HODAKA TAKEUCHI, FUMI TSUNODA, NAOKI TANAKA, TANRI SHIOZAWA

**Affiliations:** 1 Department of Obstetrics and Gynecology, School of Medicine, Shinshu University, Matsumoto, 390-8621, Japan; 2 Department of Global Medical Research Promotion, School of Medicine, Shinshu University Graduate, Matsumoto, Nagano, 390-8621, Japan; 3 International Relations Office, School of Medicine, Shinshu University, Matsumoto, Nagano, 390-8621, Japan; 4 Research Center for Social Systems, Shinshu University, Matsumoto, Nagano, 390-8621, Japan

**Keywords:** Peroxisome proliferator-activated receptor alpha, Sterol regulatory element-binding protein 1, Antioxidant, Migration

## Abstract

Endometrial carcinoma (EMC) is associated with obesity; however, the underlying mechanisms have not yet been elucidated. Peroxisome proliferator-activated receptor alpha (PPARα) is a nuclear receptor that is involved in lipid, glucose, and energy metabolism. PPARα reportedly functions as a tumor suppressor through its effects on lipid metabolism; however, the involvement of PPARα in the development of EMC remains unclear. The present study demonstrated that the immunohistochemical expression of nuclear PPARα was lower in EMC than in normal endometrial tissues, suggesting the tumor suppressive nature of PPARα. A treatment with the PPARα activator, irbesartan, inhibited the EMC cell lines, Ishikawa and HEC1A, by down-regulating sterol regulatory element-binding protein 1 (SREBP1) and fatty acid synthase (FAS) and up-regulating the tumor suppressor genes p21 and p27, antioxidant enzymes, and AT-rich interaction domain 1A (ARID1A). These results indicate the potential of the activation of PPARα as a novel therapeutic approach against EMC.

## Introduction

According to the statistics of the American Cancer Society, endometrial carcinoma (EMC) is one of the most important cancers among women [1], and its incidence has been increasing in recent years. The number of EMC patients has continued to increase, particularly in developed countries, which may be attributed to lifestyle changes and high-calorie diets, and obesity has been identified as one of the most critical risk factors for EMC [2]. Previous studies suggested the underlying mechanisms linking obesity and EMC, which include insulin resistance, adipokines, inflammation, and metabolic syndrome; however, a clear explanation has not yet been established.

Tumor cells are considered to undergo metabolic reprogramming in response to an augmented need for energy [3]. This reprogramming increases the production and consumption of metabolic intermediates during biosynthesis, which are regarded as a hallmark of cancer [4]. Well-known metabolic changes in tumors are the increased uptake and use of glucose, called the Warburg effect. In addition, tumor cells have been reported to show an increase in lipid synthesis in association with glucose metabolism [5]. Therefore, changes in lipid metabolism, particularly fatty acid (FA) synthesis and oxidation, are being increasingly regarded as essential events in tumor growth and aggressiveness.

Fatty acid synthase (FAS), which generates various FAs, is normally expressed in the liver, and is limited in other tissues. However, cancer tissues and normal tissues exhibiting strong proliferative activity, such as the normal endometrium, express higher levels of FAS [6], suggesting a relationships between FAS expression and proliferative potential. Sterol regulatory element-binding proteins (SREBPs) are membrane-bound transcription factors that belong to the basic helix-loop-helix leucine zipper family [7–9]. Recent studies revealed that sterol regulatory factor-binding protien-1 (SREBP1) is a key transcriptional factor involved in lipogenesis including FAS [10] because its binding elements (sterol response elements) are present in the promoter regions of genes for the biosynthesis of FAs, lipids, and cholesterol. A previous study reported that the expression of SREBP1 co-localized with that of FAS and the proliferation marker, Ki-67 [10]. In addition, the overexpression of SREBP1 has been detected in several cancers, such as the liver, breast, prostate, and bladder; therefore, the up-regulated expression of FAS in tumor cells is considered to be related to the overexpression of SREBP1 [11–14].

In contrast to SREBP1, peroxisome proliferator-activated receptor alpha (PPARα) is a ligand-activated, nuclear receptor/transcriptional factor that is mainly expressed in the liver, heart, kidneys, and skeletal muscle, and is involved in the activation of FA catabolism and subsequent reduction of FA/triglyceride (TG) levels by beta-oxidation [15–17]. It may also reduce the toxicity of a high cholesterol diet in the human body [18]. Although a previous study reported that fenofibrate, a PPARα activator, suppressed the proliferation of and induced apoptosis in EMC cells [19], the underlying mechanisms remains unclear. The activation of PPARα has been shown to suppress the LXR-SREBP1 pathway [20], indicating that PPARα is a potent negative regulator of SREBP1. In addition, a PPARα activator was found to reduce the nuclear expression of SREBP1 in rat liver cancer cells, and this decrease was mediated by the up-regulated expression of insulin-induced gene 1 [21], a key regulator of SREBP1. Collectively, these findings suggest that the tumor suppressor function of PPARα is mediated by a reduction in the expression of SREBP1.

In addition to SREBP1, recent studies reported the involvement of cell cycle regulators in the PPARα-induced growth suppression of tumor cells. p21 and p27 are important members of the cyclin-dependent kinase inhibitor family [22]. Previous findings demonstrated that a PPARα activator suppressed the development of colon cancer by up-regulating the expression of p21 and p27, suggesting a PPAR strategy for the prevention of colon cancer [23]. Moreover, an abnormality in the AT-rich interaction domain 1A (ARID1A) gene, an essential molecule in chromosomal stability, was shown to be closely involved in the development of EMC [24]; however, the role of ARID1A in PPARα-mediated growth regulation has not been examined.

Irbesartan (Irbe), a conventional angiotensin II receptor blocker, is frequently used as an antihypertensive drug in clinical practice. In contrast to fibrates, Irbe activates PPARα without significant adverse effects [25]. Hypertension is an important risk factor for EMC [2], and angiotensin II has been reported to promote the survival of EMC cells [26]. In addition, the activation of PPARα by fibrates was previously shown to inhibit the growth of EMC cells *in vitro* via PPARα [19]. Therefore, Irbe has potential as a therapeutic agent for EMC.

Accordingly, we hypothesized that the effects of PPARα on tumor cells may be attributed to abnormal lipid metabolism and the related expression of tumor suppressors. To test this hypothesis, we investigated the expression and function of PPARα by immunohistochemistry, gene silencing, and with the PPARα activator, Irbe, using EMC Ishikawa cells. In addition, the involvement of SREBP1, p21 and p27, and ARID1A was examined using immunoblotting and immunofluorescence.

## Materials and Methods

### Immunohistochemistry

**1) Collection of normal and neoplastic endometrial tissues**

Formalin-fixed, paraffin-embedded tissues of the normal endometrium (5 cases each of the proliferative and secretory phases) and EMC (22 cases of Grade 1 (G1), 14 of Grade 2 (G2), and 14 of Grade 3 (G3)) were collected from the pathology files of patients who underwent biopsy or hysterectomy at Shinshu University Hospital in Matsumoto City between 2020 and 2021. All participants were Japanese individuals living in Nagano prefecture, Japan. The median age of participants with a normal endometrium was 46 years (35 to 49 years, premenopausal), while that of EMC patients was 55 years (29 to 83 years). These EMC patients included 24 cases (12 in G1, 5 in G2, and 7 in G3) with a body mass index (BMI) >25, defined as obese in Japan. We used tissue sections from these patients for immunohistochemical and hematoxylin and eosin (HE) staining.

**2) Staining procedure**

**Figure S1:**
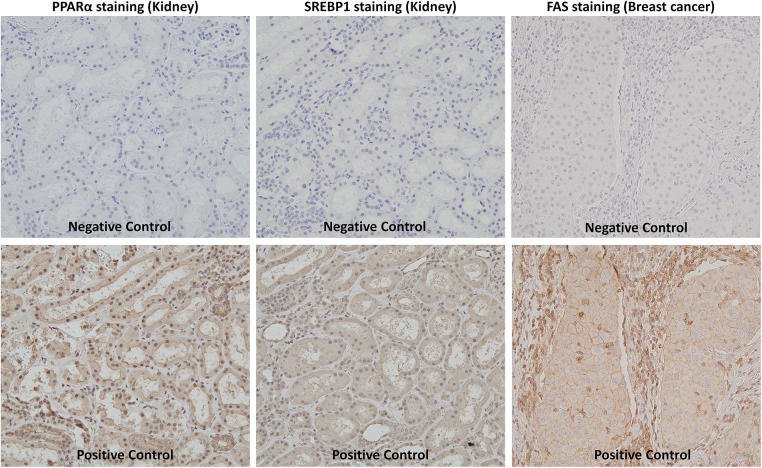
Negative and positive control of immunostaining.

We performed immunohistochemical staining as previously described [27]. Briefly, indirect immunostaining for PPARα, SREBP1, and FAS was conducted using anti-PPARα (1:100 dilution, mouse monoclonal, H-2, sc-398394, Santa Cruz Biotechnology, Dallas, TX, USA), anti-SREBP1 (1:100 dilution, rabbit polyclonal, NB100-2215, Novus Biologicals, Centennial, CO, USA), and anti-FAS (1:500 dilution, rabbit monoclonal, C18C12, #4233, Cell Signaling Technology, Danvers, MA, USA) antibodies, respectively, as primary antibodies. N-Histofine® Simple Stain Max-PO(M) and (R) (414132F and 414142F, Nichirei Biosciences Inc., Tokyo, Japan) were used as the secondary antibodies for anti-PPARα and anti-SREBP1, respectively. Three-micrometer-thick sections were deparaffinized in d-Limonene (Hemo-De®, FALMA, Tokyo, Japan) and rehydrated in a graded alcohol series. Antigens were retrieved by a microwave pretreatment in 10 mM Tris/HCl buffer (pH 8.0) containing 1 mM EDTA for 30 min. Sections were then exposed to primary antibodies at 4°C overnight. As a positive control, we used kidney tissue sections for anti-PPARα and anti-SREBP1 antibodies and breast cancer tissue sections for anti-FAS antibodies (Suppl. Fig. S1). As a negative control, tissue sections were treated with a buffered solution without the primary antibody. These sections were then incubated with the secondary antibody at room temperature for 60 min. The immunocomplex was visualized by diaminobenzidine (349-00903, FUJIFILM Wako Pure Chemical, Osaka, Japan).

**3) Staining evaluation**

The immunoreactivity of each stain was assessed by the Histo Score (H-score). We took three microscopic photographs per immunostaining slide at ×200 magnification and automatically counted nuclei with each nuclear staining intensity (strong, moderate, weak, and no stain) using free ImageJ software (https://imagej.nih.gov/ij/). Nuclear staining intensity was graded as follows: 0 = no stain (similar to the negative control), 1 = weak stain, 2 = moderate stain (equivalent to the positive control), and 3 = strong stain. The H-score was calculated using the following formula: [1 × (% nuclei with weak staining) + 2 × (% nuclei with moderate staining) + 3 × (% nuclei with strong staining)]. The final score ranged between 0 and 300. Cytoplasmic staining for FAS was also automatically counted using ImageJ and evaluated by the H-score.

### Cell culture

**Figure S2:**
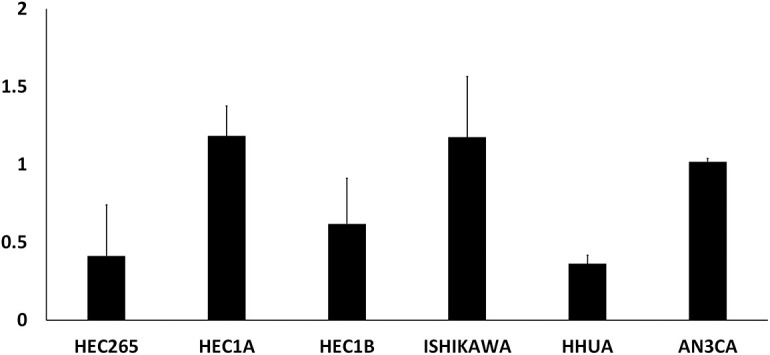
Relative expression of PPARα mRNA in EMC cell lines.

EMC cells: The ERα-positive EMC cell line, Ishikawa 3-H-12 (Ishikawa), was kindly gifted from Dr. Nishida (Kasumigaura Medical Center, Tsuchiura, Japan) through the Japanese Collection of Research Bioresources (JCRB) Cell Bank (JCRB1505, Ibaraki, Japan). The ERα-negative EMC cell line, HEC1A, was purchased from the American Type Culture Collection (HTB-112, Manassas, VA, USA). Among the EMC cell lines we had, these two showed high PPARα expression (Suppl. Fig. S2). Ishikawa cells were cultured in Dulbecco’s modified Eagle medium (D6046, Sigma-Aldrich, St. Louis, MO, USA) supplemented with 15% fetal bovine serum (FBS) (10270-106, Thermo Fisher Scientific, Waltham, MA, USA). HEC1A cells were cultured in McCoy’s 5A medium (M9309, Sigma-Aldrich) supplemented with 10% FBS. Cells were incubated at 37°C in a 5% CO_2_ incubator.

### siRNA transfection

Ishikawa and HEC1A cells were transfected with siRNA as previously described [28]. In brief, on the day before use in experiments, cells were seeded on 6-well plates at a density of 2 × 10^5^ cells/well. After 24 h, cells in the siPPARα group were treated with 5 nM siRNA for PPARα (CCUUGCAGGCCACUCGAGCCCUAAU) (Stealth RNAi^™^ siRNA, Thermo Fisher Scientific). Lipofectamine RNAiMAX Reagent (Cat. No. 13778030, Thermo Fisher Scientific) was used for siRNA transfection according to the manufacturer’s instructions. The negative control (NC) group received Stealth RNAi^™^ siRNA negative control lo GC (12935-200, Thermo Fisher Scientific). Cells were then used in subsequent experiments. After transfection for 72 h, cells were harvested to confirm silencing by Western blotting and RT-PCR.

### Agents

Irbe, used in experiments as an inducer of PPARα expression, was purchased from Sigma-Aldrich (Cas No. 138402-11-6) and dissolved in dimethyl sulfoxide (DMSO) (FUJIFILM Wako Pure Chemical) for addition to cultured cells. Cells in the Irbe group were treated with 100 µM Irbe. This concentration was selected based on previous studies [25,29]. Cells in the NC group were treated with DMSO only.

### Cell proliferation assay

The WST-1 assay was performed to examine the effects of PPARα on cell proliferation using Cell Proliferation Reagent WST-1 (Code: 11644807001, Roche Diagnostics, Basel, Switzerland) and a microplate reader (BioTek Synergy HTX Multimode Reader: Agilent Technologies, Santa Clara, CA, Japan). As previously described [28], cells were seeded on a 96-well microplate at a density of 1000 cells/well. According to the manufacturer’s instructions, the WST-1 assay was performed for 4 consecutive days after the confirmation of cell attachment to the bottom of the wells. All experiments were conducted using eight wells for each condition and repeated three or more times to confirm reproducibility.

### Scratch wound healing assay

The effects of PPARα on migration were analyzed by the scratch wound healing assay using Ishikawa and HEC1A cells and then compared between PPARα siRNA (siPPARα) and NC siRNA (NC) or between Irbe and DMSO (NC). Cells were prepared as previously described [30]. In brief, 8.2 × 10^4^ cells were plated onto 6-well plates and cultured under each condition, such as siRNA transfection or the Irbe treatment. The surface of the plates containing cells at 70% confluence was scratched linearly with a new 200-μl pipette tip to form the gap recorded by photographs (0 h, ×200 magnification by phase contrast observations). After 48 h, we photographed the same gap positions (48 h). ImageJ software automatically measured the area occupied by migrated cells in the gap formed by the scratch at 48 h in these photographs. We obtained results from 3 independent experiments with three or more measurement points.

### Western blotting

Protein expression levels in Ishikawa and HEC1A cells were examined by Western blotting, as previously described [28]. In brief, 8.2 × 10^4^ EMC cells were cultured in a 6-well plate. Cells were harvested 72 h after gene silencing and drug treatment and lysed in 100 µL of lysis buffer. The supernatant protein was extracted by −80°C quick freezing and thawing on ice. Protein concentrations were measured using the Pierce^™^ BCA Protein Assay kit (Cat: 23227, Thermo Fisher Scientific). The nuclear and cytoplasmic fractions of cell extracts were prepared using NE-PER^™^ Nuclear and Cytoplasmic Extraction Reagent (Thermo Fisher Scientific). Immunoblotting was performed using antibodies targeting PPARα (1:200, sc-398394), SREBP1 (1:100, NB100-2215), FAS (1:1000, C18C12, #4233), the cell cycle-dependent kinase inhibitors p21 (1:500, mouse monoclonal, Cat. No: 554228, BD Pharmingen, San Diego, CA, USA) and p27 (1:200, mouse monoclonal, sc-1641, Santa Cruz Biotechnology), superoxide dismutase 1 (SOD1, 1:5000, mouse monoclonal, sc-17767, Santa Cruz Biotechnology) superoxide dismutase 2 (SOD2, 1:1000, rabbit monoclonal, D9V9C, #13194, Cell Signaling Technology), glutathione peroxidase 1 (GPx1, 1:200, mouse monoclonal, sc-22146, Santa Cruz Biotechnology), NADH quinone oxidoreductase (NQO1, 1:1000, rabbit polyclonal, Cat. No. GTX100235, GeneTex, Irvine, CA, USA), and ARID1A (1:1000, rabbit polyclonal, HPA005456, Sigma-Aldrich). The bands of β-actin (1:1000, mouse monoclonal, A5441, Sigma-Aldrich) and Lamin B1 (1:2000, mouse monoclonal, NA-12-100UG, Oncogene, Boston, MA, USA) were used as the loading controls of whole-cell lysates and nuclear fractions, respectively. Protein-transferred membranes were incubated with the specific primary antibody at 4°C overnight and then with the secondary antibody at room temperature for 1 h. The target protein band bound by the antibody complex was then visualized using the ECL Western blot detection reagent (RPN-2109, Amersham, Amersham, UK). Image J software was used to analyze band density. Band densities were calculated relative to the density of β-actin and Lamin B1 expression. All experiments were repeated three or more times to confirm reproducibility.

### Real-time quantitative RT-PCR (qPCR) analysis

The expression of mRNA in Ishikawa cells was analyzed by real-time qPCR. The total RNA of cells cultured on 6-well plates under specific conditions was isolated using TRIzol reagent (Cat. No. 15596026, Thermo Fisher Scientific). Reverse transcription (RT) and qPCR were performed using the PrimeScript RT-PCR kit (RR014B, Takara Bio, Shiga, Japan) according to the manufacturer’s instructions. Primer sequences are listed in Suppl. Table S1. The mRNA expression levels of each gene were corrected by the mRNA expression level of ACTB, the endogenous control, and shown as a relative value to NC. Experiments were repeated three times with four replicates.

### Reactive oxygen species (ROS) assay

We investigated the effects of the Irbe treatment on intracellular ROS production in Ishikawa cells. A total of 5 × 10^3^ cells/well of Ishikawa cells were plated on 96-well plates. After cells had attached to the bottom of the well, they were treated with 100 µM Irbe (the Irbe group) or DMSO only (the NC group) for 24 h. Intracellular ROS production was measured using a Fluorometric Intracellular ROS Kit (MAK143, Sigma-Aldrich) according to the manufacturer’s instructions. Experiments were repeated three times with eight replicates.

### Immunofluorescent staining analysis

We performed dual immunofluorescent staining for PPARα and SREBP1, p21, or p27 in Irbe-treated (Irbe group) and DMSO-treated (NC group) Ishikawa cells, as previously described [31]. We used a mouse monoclonal anti-PPARα antibody (working concentration 4 µg/mL, sc-398394, Santa Cruz Biotechnology) and Alexa Fluor® 594-conjugated goat anti-mouse IgG antibody (red-fluorescence, A-11005, Thermo Fisher Scientific) for PPARα. We used rabbit polyclonal primary antibodies (anti-SREBP1: working concentration 4 µg/mL, NB100-2215, Novus Biologicals; anti-p21: working concentration 0.2 µg/mL, sc-756, Santa Cruz Biotechnology; anti-p27: working concentration 0.2 µg/mL, sc-528, Santa Cruz Biotechnology) and an Alexa Fluor® 488-conjugated goat anti-rabbit IgG antibody (green-fluorescence, A-11008, Thermo Fisher Scientific) for SREBP1, p21, and p27.

A total of 2 × 10^4^ Ishikawa cells/well were cultured in 8-well chamber slides and treated for 24 h. Cells were washed once with phosphate-buffered saline (PBS) and then fixed with 4% paraformaldehyde phosphate buffer (09154-56, Nacalai Tesque, Kyoto, Japan) for 15 min. After washing with PBS, cells were blocked with 1% bovine serum albumin (Sigma-Aldrich) in PBS for one hour at room temperature, the primary antibody was added, and cells were then incubated at 4°C overnight. After washing with PBS, cells on slides were covered with the secondary antibody solution and set in the dark for one hour. After washing with PBS, cells were nuclear-counterstained by DAPI (D9542, Sigma-Aldrich) and sealed with Fluorescence Mounting Medium (S3023, DAKO, Glostrup, Denmark). All specimens were observed at ×200 magnification using the Zeiss LSM880 confocal imaging system (Carl Zeiss, Oberkochen, Germany). We used ImageJ software to measure the immunofluorescent signal intensity of photographs. Experiments were repeated three times.

### Statistical analysis

All data are presented as the mean ± standard deviation. The results of immunostaining of the endometrium were evaluated using Scheffé’s method, and other comparisons of two groups were conducted using the Student’s *t*-test. *p* < 0.05 was considered to be significant.

## Results

### PPARα expression decreased, while SREBP1 and FAS expression reciprocally increased in EMC tissues

**Figure 1:**
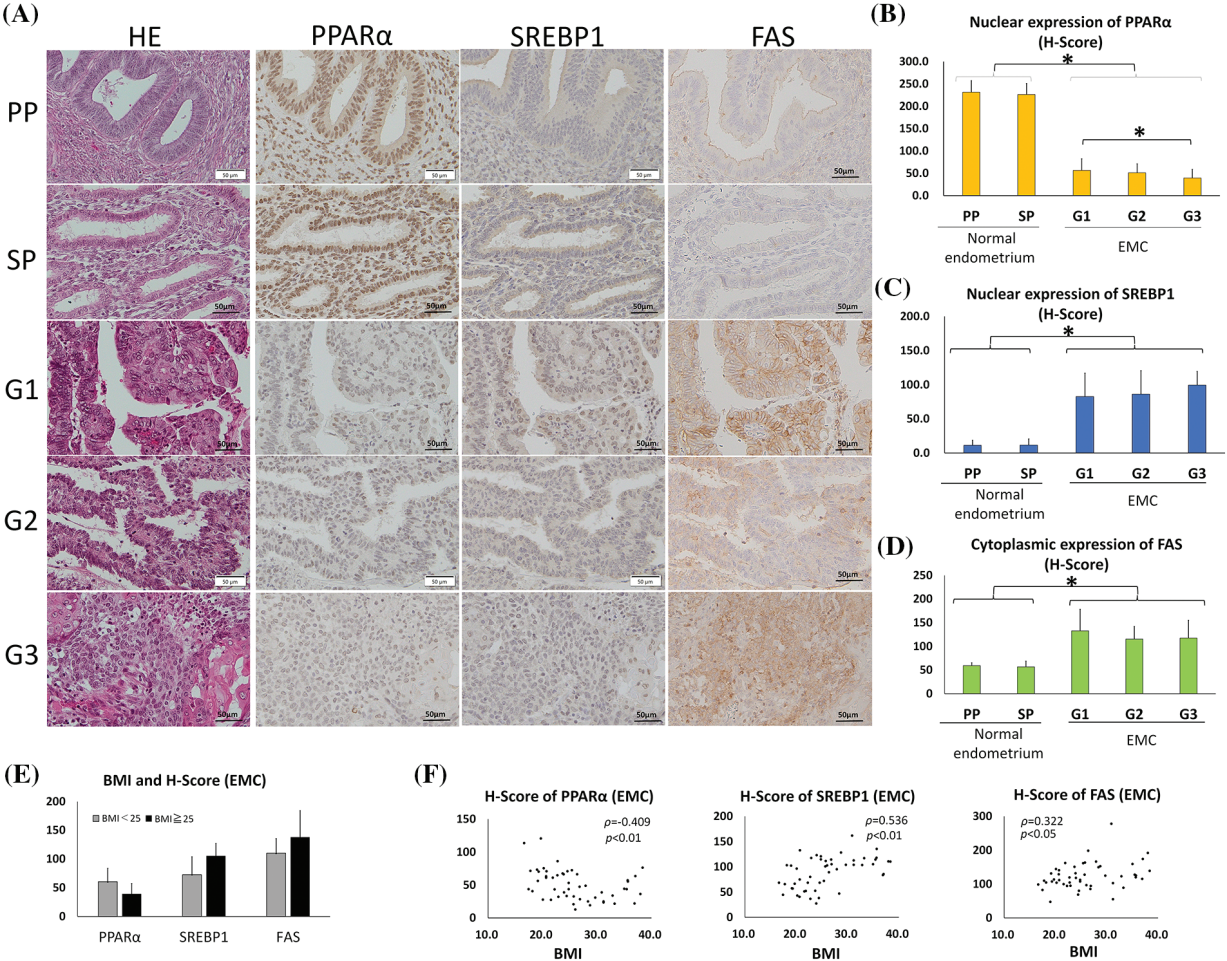
PPARα expression was lower and SREBP1 and FAS expression was higher in endometrial carcinoma (EMC) than in normal endometrial glands. (Immunohistochemistry). (A) Photomicrographs show hematoxylin and eosin (HE) staining and representative immunostaining for PPARα, SREBP1, and FAS in serial sections of the normal endometrium (PP: proliferation phase; SP: secretory phase) and EMC (G1: grade 1; G2: grade 2; G3: grade 3). (B–D) Graphic demonstration of immunostaining for PPARα (B), SREBP1 (C), and FAS (D). The expression of PPARα was significantly lower in EMC than in the normal endometrium, while the expression of SREBP1 and FAS was significantly higher (* *p* < 0.01). (E) The graph indicates the H-scores of PPARα, SREBP1, and FAS in non-obese EMC patients with BMI <25 (*n* = 26) or in obese EMC patients with BMI >25 (*n* = 24). There was no significant difference. (F) The BMI and H-scores of PPARα, SREBP1, and FAS in each EMC patient are shown in scatter plots. Spearman’s rank correlation coefficient showed a weak inverse correlation between BMI and the PPARα H-score and a moderate or weak correlation between BMI and SREBP1 or FAS.

Since PPARα is a nuclear receptor, we considered the nuclear staining of PPARα to be significant in the normal endometrium (Figs. 1A and 1B). The H-scores of nuclear PPARα expression in endometrium epithelia were 231 ± 26 (mean ± SD) in the proliferative phase and 226 ± 25 in the secretory phase, with no significant difference. The H-scores of nuclear PPARα expression in G1, G2, and G3 tumors were 56 ± 26, 51 ± 20, and 39 ± 19, respectively, with a significant difference between G1 and G3. However, the H-score of nuclear PPARα was significantly lower in all EMC tumors than in normal endometrium epithelia (*p* < 0.01). SREBP1 is an inactive precursor that binds to the endoplasmic reticulum. It is transported from the endoplasmic reticulum to the Golgi apparatus, in which it is activated. Activated SREBP1 is then transferred to the nucleus to synthesize cholesterol and FAs [8, 32, 33]. The H-scores of nuclear SREBP1 expression in the proliferative and secretory phases of endometrium epithelia were 11 ± 7 and 12 ± 9, respectively, with no significant difference (Figs. 1A and 1C). The H-scores of nuclear SREBP1 expression in G1, G2, and G3 tumors were 83 ± 34, 86 ± 34, and 100 ± 20, respectively, with no significant differences (Figs. 1A and 1C).

However, the H-score of nuclear SREBP1 was significantly higher in all EMC tumors than in the normal endometrium (*p* < 0.01). Similar to SREBP1, the H-score of cytoplasmic FAS was significantly higher in EMC than in normal endometrial epithelia (*p* < 0.01) (Figs. 1A and 1D). These results indicate that as PPARα expression decreased, SREBP1 and FAS reciprocally increased in EMC.

In this EMC case series, we compared the expression of PPARα, SREBP1, and FAS between obese Japanese patients with BMI >25 (*n* = 24) and non-obese patients with BMI <25 (*n* = 26), but observed no significant difference (Fig. 1E). However, a weak correlation was noted between BMI and the H-scores of PPARα (inverse correlation), SREBP1, and FAS by Spearman’s rank correlation (Fig. 1F).

### PPARα suppresses the proliferation and migration of EMC cells

**Figure 2:**
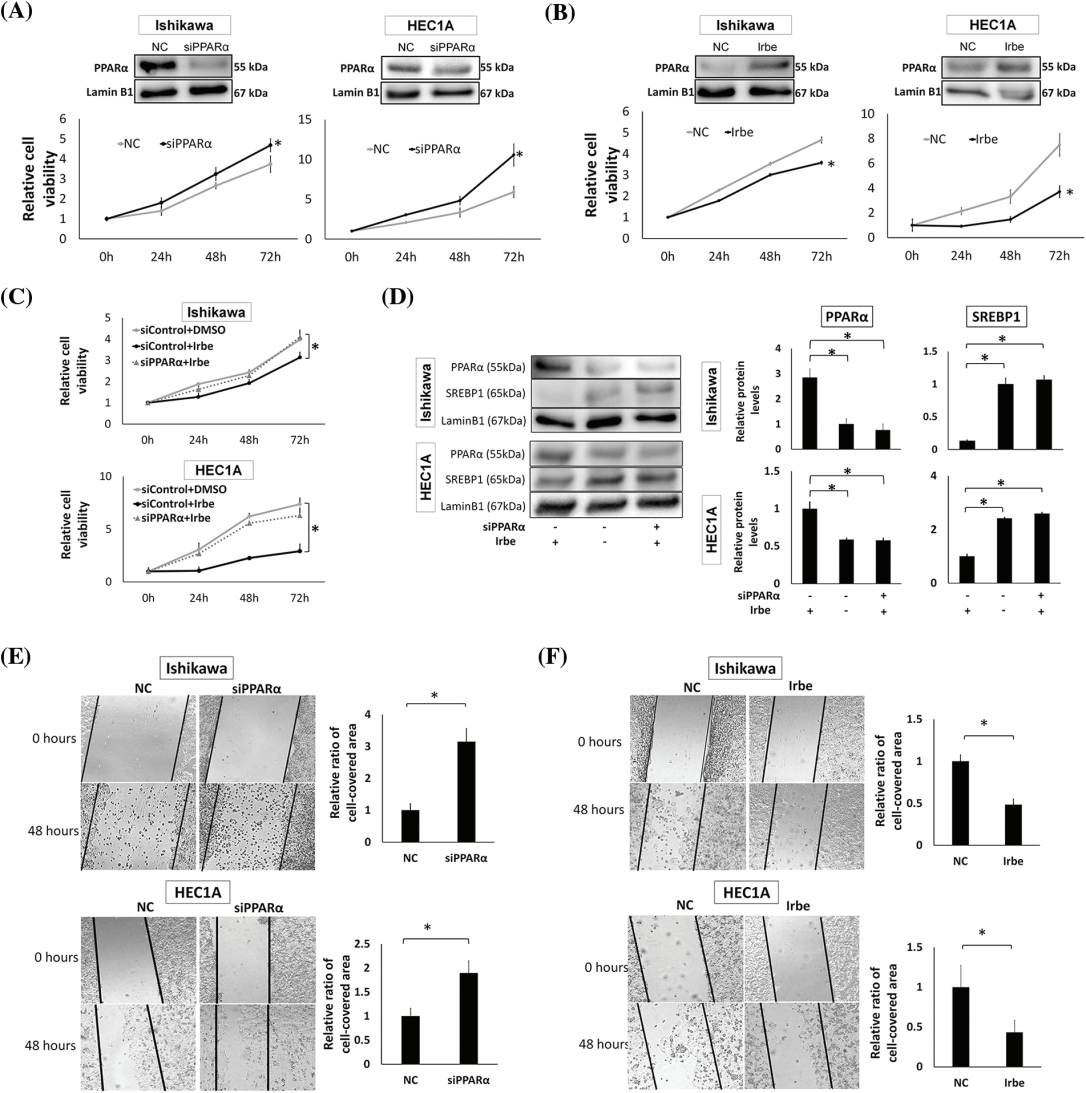
PPARα reduces the proliferation and migration of EMC cell lines, Ishikawa and HEC1A. (A) PPARα suppression by siRNA enhanced the proliferation of Ishikawa and HEC1A cells. Western blotting shows that PPARα protein expression was significantly lower than that in the negative control (NC) after the treatment with PPARα siRNA (siPPARα) (*p* < 0.05). The WST-1 assay indicates that siPPARα induced significant increases in proliferation at 72 h (h). (B) Western blotting and the WST-1 assay show that PPARα protein expression was significantly higher (*p* < 0.05), while Ishikawa and HEC1A cell proliferation was lower with the addition of Irbesartan (Irbe) (100 µM) than with the NC at 72 h. (C) The Irbesartan (Irbe) treatment suppressed the proliferation of Ishikawa and HEC1A cells by up-regulating the expression of PPARα. Viability was lower in cells treated with the Irbe treatment (siControl+Irbe) than in the untreated negative control (siControl+DMSO) at 72 h. However, this effect of Irbe was almost entirely canceled by the suppression of PPARα by siRNA (siPPARα+Irbe). (D) Western blotting showed that PPARα expression was higher and SREBP1 expression was lower in both EMC cells after the Irbe treatment than in the untreated control. This effect of Irbe was canceled by the suppression of PPARα by siRNA (siPPARα). (E) In comparisons with the NC, the scratch wound healing assay indicated that PPARα silencing by siRNA (siPPARα) significantly facilitated the migration of Ishikawa and HEC1A cells. (F) The scratch assay also showed that the Irbe treatment (Irbe) reduced migration. * *p* < 0.05.

To investigate the effects of PPARα on the growth of EMC cells, we examined the effects of PPARα silencing using the EMC cell lines, Ishikawa and HEC1A. We found that PPARα silencing significantly increased the proliferation of both cell lines at 72 h (Fig. 2A). The addition of Irbe significantly suppressed the growth of Ishikawa and HEC1A cells at 72 h (Fig. 2B). The growth suppressive effects of Irbe (siControl+Irbe, Fig. 2C) relative to untreated NC cells (siControl+DMSO) were almost fully canceled by PPARα silencing (siPPARα+Irbe) in both EMC cell lines (Fig. 2C). Therefore, we considered Irbe to exert anti-proliferative effects by activating PPARα. Immunoblotting results also showed that the Irbe treatment (siControl+Irbe) up-regulated the protein expression of PPARα and down-regulated that of SREBP1 (Fig. 2D). This effect of Irbe was canceled by the inhibition of PPARα expression (siPPARα+Irbe, Fig. 2D). These results demonstrated that Irbe suppressed the proliferation of EMC cells by up-regulating PPARα expression.

The effects of PPARα on cell migration were examined using the scratch wound healing assay. In comparisons with the NC, migration activity increased after the PPARα gene was silenced (Fig. 2E), and the Irbe treatment up-regulated PPARα expression and significantly decreased migration (Fig. 2F). These results indicate that the activation of PPARα suppressed the proliferation and migration of EMC cells. In the experiments shown in Fig. 2, HEC1A and Ishikawa cells exhibited similar changes in PPARα and SREBP1 protein expression and cellular functions, such as proliferative and migratory abilities, in response to PPARα silencing and the Irbe treatment. Therefore, we used Ishikawa cells in subsequent experiments.

### PPARα suppresses SREBP1 and FAS expression and enhances p21 and p27 expression

**Figure 3:**
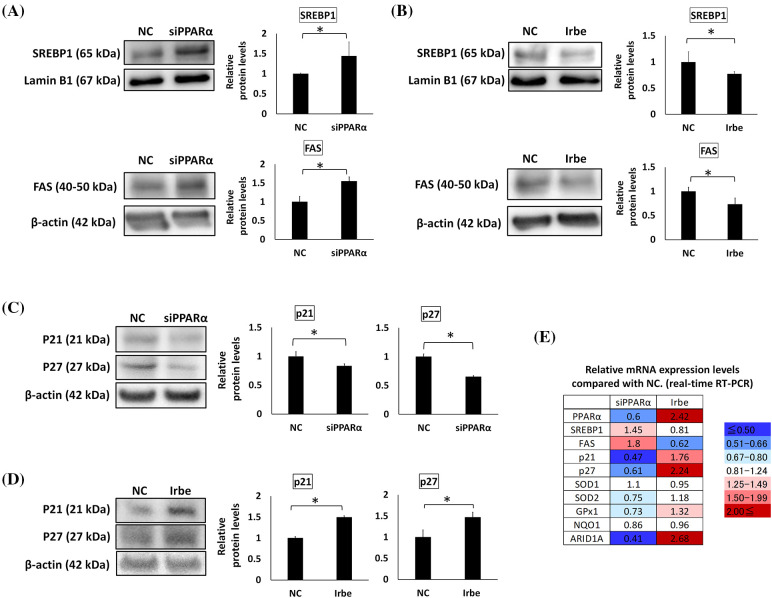
PPARα suppresses SREBP1 and FAS expression and promotes p21 and p27 expression. (A–D) Western blotting. (E) Real-time RT-qPCR. (A) PPARα gene silencing (siPPARα) increased SREBP1 and FAS protein expression over that in the negative control (NC). (B) The PPARα inducer, Irbe (100 µM) reduced SREBP1 and FAS protein expression to lower than that in the NC. (C) PPARα gene silencing suppressed p21 and p27 protein expression. (D) The Irbe (100 µM) treatment increased p21 and p27 protein expression. (E) The mRNA expression level of each gene after PPARα silencing (siPPARα) and the Irbe treatment (Irbe) was measured by real-time RT-qPCR and shown as a relative value to NC. The heat map shows expression reductions in blue and expression increases in red. Although the fold change in SREBP1 was smaller than that in FAS, SREBP1 and FAS mRNA expression inversely correlated with PPARα. On the other hand, the mRNA expression of p21, p27, SOD1, and GPx1 correlated with PPARα. NC: negative control. * *p* < 0.05.

The effects of PPARα on the expression of SREBP1, FAS, and p21/p27 in Ishikawa cells was investigated by gene silencing and the Irbe treatment. The expression of SREBP1 and FAS was increased by PPARα silencing and decreased by the addition of Irbe, indicating an inverse correlation between the expression of PPARα and SREBP1/FAS proteins (Figs. 3A and 3B). The expression of the p21 and p27 proteins was decreased by PPARα silencing and increased by the Irbe treatment, indicating a positive correlation between the expression of PPARα and p21/p27 (Figs. 3C and 3D).

The mRNA expression levels of these genes were also examined using real-time qPCR. Expression levels compared with the NC are shown in Fig. 3E. Although fold changes in SREBP1 mRNA expression from that in the NC were small, the results obtained suggested that SREBP1 and FAS mRNA expression may both inversely correlate with PPARα mRNA expression, similar to protein expression. Changes in p21 and p27 expression levels also correlated with those in PPARα expression levels, similar to protein expression (Fig. 3E).

### PPARα promotes antioxidant functions and ARID1A expression in EMC cells

**Figure 4:**
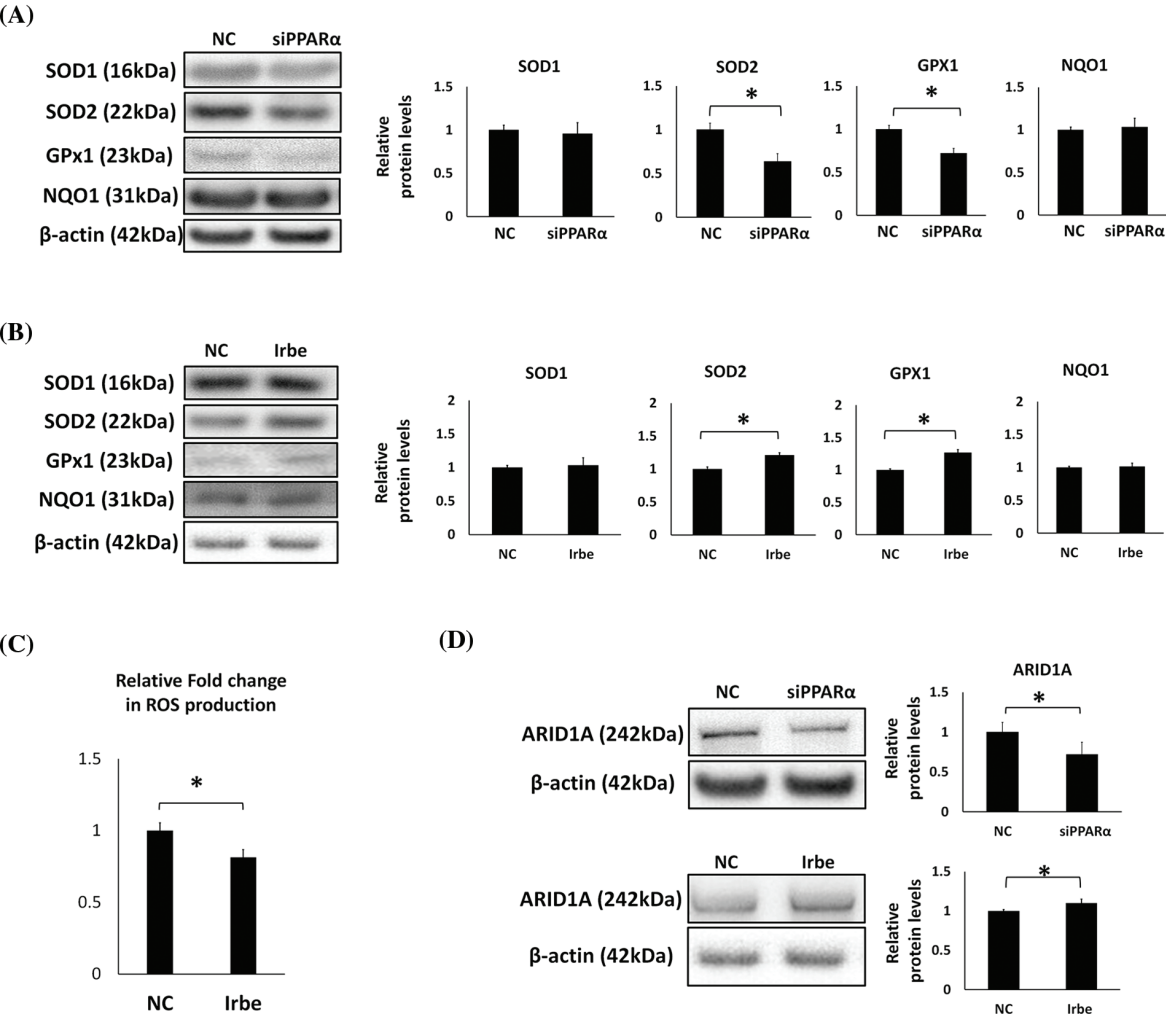
PPARα promotes the expression of antioxidant enzymes, SOD2 and GPx1, and the vital tumor suppressor factor, ARID1A, in Ishikawa cells. (A) PPARα gene silencing decreased SOD2 and GPx1 protein expression. (B) The Irbe (100 µM) treatment increased SOD2 and GPx1 protein expression. (C) The graph shows intracellular reactive oxygen species (ROS) production measured by the ROS assay in Ishikawa cells treated with 100 µM Irbesartan (Irbe) for 24 h or DMSO (NC). This result indicates that Irbe reduced intracellular ROS production in EMC cells. (D) PPARα gene silencing decreased, while the Irbe (100 µM) treatment increased ARID1A protein expression. * *p* < 0.05.

Oxidative stress is an important factor in the formation and development of cancer [34]. Since previous studies demonstrated that PPARα exerts antioxidant effects [35], we examined the effects of PPARα silencing and the addition of Irbe on the expression of the typical antioxidant enzymes, SOD1, SOD2, GPx1, and NQO1, in Ishikawa cells. Real-time RT-qPCR suggested that the mRNA expression of SOD2 and GPx1 correlated with that of PPARα (Fig. 3E). These results indicated that PPARα silencing reduced SOD2 and GPx1 protein expression (Fig. 4A). In contrast, the Irbe treatment increased SOD2 and GPx1 protein expression (Fig. 4B). In addition, the results of the ROS assay suggested that the Irbe treatment attenuated intracellular ROS production in Ishikawa cells (Fig. 4C). Therefore, PPARα may ameliorate oxidative stress in EMC Ishikawa cells.

Since ARID1A is regarded as an important tumor suppressor in EMC, the effects of PPARα silencing and activation on the expression of ARID1A were examined. The results obtained indicated that PPARα silencing reduced, while the addition of Irbe increased ARID1A protein expression (Fig. 4D). These results suggest the involvement of PPARα in ARID1A-mediated oncogenic suppression in endometrial cancer cells.

### The PPARα activator, Irbe, reduces SREBP1 and increases p21/p27 in PPARα-activated cells

**Figure 5:**
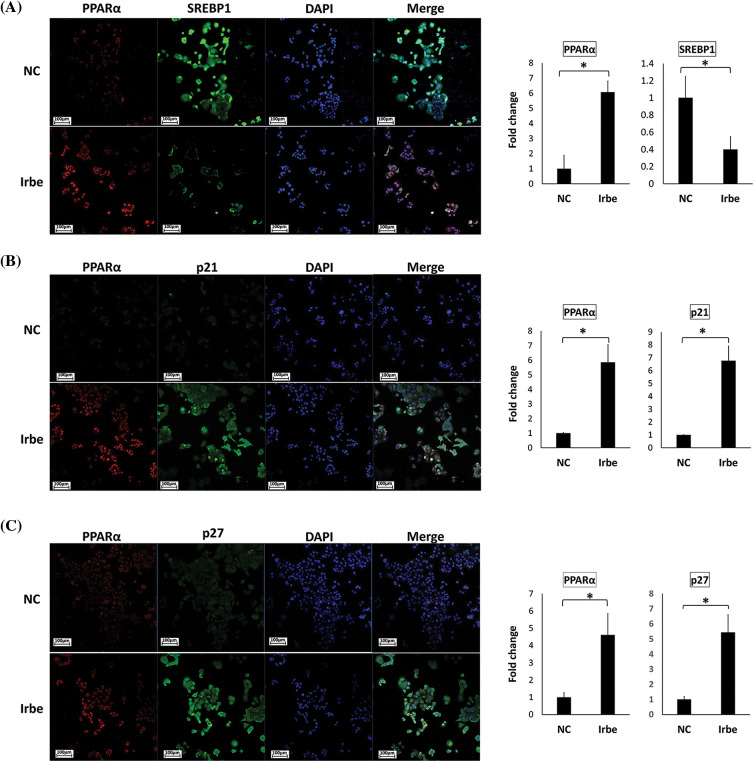
The PPARα activator, Irbe, reduces SREBP1 expression and induces the expression and co-localization of PPARα and p21/p27 in Ishikawa cells. (A) Dual immunofluorescent staining for PPARα (red) and SREBP1 (green) show that the Irbe (100 µM) treatment increased the nuclear expression of the PPARα protein while decreasing that of SREBP1. (B, C) Dual immunofluorescent staining for PPARα (red) and p21 (B) or p27 (C) (green) showed that the Irbe treatment increased the expression of both proteins, and their distribution was almost consistent. *p < 0.05.

To examine changes in the subcellular distribution of PPARα, SREBP1, and p21/p27 proteins in Ishikawa cells treated with Irbe, immunofluorescent staining was performed. The PPARα signal was observed in nuclei, and the number of PPARα-positive cells was higher after the administration of Irbe than in the NC group (Figs. 5A–5C).

In contrast, SREBP1-positive cells in the nucleus decreased in the Irbe-treated group (Fig. 5A). On the other hand, the Irbe treatment increased the expression of p21 and p27 (Figs. 5B and 5C). Furthermore, the distribution of PPARα-positive and p21/p27-positive cells was similar to that in the Irbe group (Figs. 5B and 5C). These results were consistent with those of immunoblotting. Since activated PPARα is stabilized in nuclei and enhances the transcriptional activity of target genes, increases in PPARα-positive cells in nuclei indicate the sufficient activation of PPARα by the Irbe treatment.

## Discussion

EMC is strongly associated with obesity; however, the underlying mechanisms remain unclear. In the present study, we demonstrated that the expression of PPARα, a pivotal transcription factor for lipid metabolism, was lower in the nuclei of EMC cells than in normal endometrial cells. In EMC tissues, the expression of PPARα inversely correlates with BMI, while that of SREBP1 and its target gene, FAS, positively correlates with BMI. Although these correlations are weak, these proteins have been implicated in the development of obesity-associated EMC. Furthermore, the function of PPARα was evoked via the down-regulation of SREBP1 and FAS. Irbe also significantly suppressed the proliferation of EMC cells through the activation of PPARα. These results indicate that PPARα functions as a tumor suppressor, and, thus, a PPARα activator may be a novel promising therapeutic agent against EMC.

The present study showed that the immunohistochemical expression of PPARα in normal endometrial glands was similar between the proliferative and secretory phases, suggesting that PPARα expression is independent of estrogen and progesterone. In normal human tissues, PPARα is abundantly expressed in the liver, heart, kidneys, and skeletal muscle. Detectable PPARα was also reported in the intestines, pancreas, lungs, and placenta [36]. PPARα in the human liver may effectively induce the expression of numerous genes involved in lipid, lipoprotein, and sugar metabolism [37]. However, the function of PPARα in other PPARα-expressing tissues, including the normal endometrium, has not yet been elucidated in detail. Recent studies revealed that PPARα has anti-inflammatory [38,39] and anti-oxidant stress functions [40]. The normal endometrium exhibits high anti-inflammatory [39] and antioxidant activities to maintain fertility [41]. Further studies are needed to clarify the function of PPARα in the normal endometrium.

The nuclear expression of PPARα was lower in EMC cells than in the normal endometrium. In EMC, the expression of PPARα was significantly lower in G3 than in G1. This result was inconsistent with previous findings [42,43], which showed stronger PPARα staining in EMC tissues. In these studies, positive PPARα staining was mainly observed in the cytoplasm and perinuclear area, and a detailed quantitative evaluation of nuclear PPARα expression was not performed. Regarding colon cancer [44] and ampullary cancer [45], the expression of PPARα was higher in cancer tissues than in their normal counterparts, which is inconsistent with the present pattern, whereas its expression was lower in mouse lung cancer [46]. Therefore, the higher or lower expression of PPARα in neoplastic tissues than in normal tissues may depend on the tissue context.

The immunohistochemical expression of SREBP1 and its target gene, FAS, was significantly higher in EMC than in normal endometrial glands, which was in contrast to that of PPARα. This result is consistent with previous findings supporting the carcinogenic nature of SREBP1 [47]. Collectively, the results of immunostaining for PPARα and SREBP1/FAS in normal and malignant endometrial tissues revealed an inverse correlation. Therefore, PPARα may regulate tumor cell proliferation by interfering with the expression of SREBP1 and FAS.

The mechanisms underlying the down-regulation of PPARα and up-regulation of SREBP1 in EMC tissues currently remain unclear. Several genetic changes in EMC, such as mutations in PTEN, PIK3CA, and microsatellite instability are reportedly involved in endometrial carcinogenesis [48,49]. However, a direct link between these mutations and the expression of PPARα/SREBP1 has not yet been reported. An endocrinological environment may contribute to this result, i.e., a previous study reported that the expression of androgen receptor was higher in endometrial tissues than in the normal endometrium [50], while another study showed that serum levels of testosterone were higher in patients with EMC than in control women [51]. In addition, the prenatal exposure of fetal mice to testosterone resulted in the decreased expression of PPARα in newborns. These findings may explain the reduced expression of PPARα in EMC tissues.

In the present study, we used Irbe to activate PPARα in EMC cells because it is superior to other peroxisome activators (fibrates) for organ damage [52,53]. To the best of our knowledge, this is the first study to examine the effects of Irbe on EMC cells. The results obtained revealed that Irbe increased the expression of PPARα and suppressed the proliferation and migration of EMC, Ishikawa, and HEC1A cells. According to the PPARα activation and silencing experiment in this study, the relationship between PPARα and SREBP1/FAS was consistent with immunostaining. Previous studies showed that SREBP1 is an accelerating factor for the tumorigenesis of EMC [47]. Therefore, PPARα may functionally inhibit the occurrence of EMC by inhibiting the SREBP1 pathway in the normal endometrium. In addition, the cell cycle regulators p21 and p27, which are closely related to tumor suppression, may participate in the suppression of EMC in association with PPARα. The loss of PPARα in the intestines has been proposed to increase DNMT1 (methyltransferase 1)-mediated p21 methylation and PRMT6 (protein arginine methyltransferase 6)-mediated p27 methylation, thereby promoting the development of colon cancer. In addition, an activator of PPARα reduced the expression of DNMT1 and PRMT6 [23]. Therefore, PPARα may play a role in EMC through the DNMT1/PRMT6-p21/p27 regulatory pathway. Another interesting result in the present study was that the expression of ARID1A, a key subunit of the SWI/SNF complex and a common tumor suppressor gene frequently mutated in gynecologic cancers [54,55], positively correlated with the expression of PPARα. A recent study reported that the antioxidant enzyme, SOD2, reduced the expression of the ARID1A gene [56]. In our experiments, the oxidative stress markers SOD2 and GPx1 were both affected by PPARα gene silencing and the Irbe treatment, and this pattern was similar to that of ARID1A. Therefore, PPARα may affect the expression of ARID1A by regulating oxidative stress. Collectively, these results suggest that the anti-tumor function of PPARα involved not only SREBP1, but also p21/p27, antioxidant enzymes, and ARID1A.

In addition, Irbe has strong advantages for systemic use in EMC patients. EMC closely correlated with obesity and elevated serum TG levels [57,58]. Hypertriglyceridemia is a risk factor for EMC [59,60]. A previous study reported that fenofibrate, an activator of PPARα, induced typical changes in serum lipids, including reductions in serum TG and increases in high-density lipoprotein cholesterol (HDL-c) [61]. Another study showed that the TG/HDL-c ratio may be a useful biological indicator for the treatment of EMC [62]. Moreover, PPARα is closely related to obesity and insulin resistance [63–65]. Animal experiments revealed that PPARα activators significantly attenuated type II insulin resistance [66,67]. Insulin resistance is an essential factor for metabolic syndrome and obesity-related carcinogenesis, including EMC [68–71]. In addition, a previous study demonstrated that the amount of insulin was higher in EMC patients than in a normal group [72]. Therefore, a reduction of PPARα may cause insulin resistance and promote EMC.

In conclusion, the present study clearly showed that the expression of PPARα, SREBP1, and FAS played important roles in the development of EMC, including the regulation of EMC cell proliferation and migration. In addition, the activation of PPARα by Irbe may inhibit the proliferation of EMC cells by regulating SREBP1 and FAS, the tumor suppressor genes p21 and p27, and the ARID1A pathway mediated by oxidative stress. These results indicate the therapeutic potential of Irbe against EMC.

## Data Availability

The datasets generated and/or analyzed during the present study are available from the corresponding author upon reasonable request.

## Supplementary Materials

**Table S1:** Primer sequences

Gene name	Primer name	Sequence
PPARα	PPARα-F	CGTCCTGGCCTTCTAAACGT
	PPARα-R	GCATCCGACTCCGTCTTCTT
SREBP1	SREBP1-F	GATGCGGAGAAGCTGCCTAT
	SREBP1-R	TTGCGCAAGACAGCAGATTT
FAS	FAS-F	TGCGTGGCCTTTGAAATGTG
	FAS-R	CTCCATGTCCGTGAACTGCT
p21(CDKN1A)	p21-F	ATGTGCACGGAAGGACTTTGT
	p21-R	ATGTAGAGCGGGCCTTTGAG
p27(CDKN1B)	p27-F	GGGGTCTGTGTCTTTTGGCT
	p27-R	AGACACTCGCACGTTTGACA
SOD1	SOD1-F	AAAGATGGTGTGGCCGATGT
	SOD1-R	CAAGCCAAACGACTTCCAGC
SOD2	SOD2-F	GCCTACGTGAACAACCTGAAC
	SOD2-R	TGGAATAAGGCCTTTGGGTTCT
GPX1	GPX1-F	AGTCGGTGTATGCCTTCTCG
	GPX1-R	TCTTGGCGTTCTCCTGATGC
NQO1	NQO1-F	CCTTGTGATATTCCAGAGTGGC
	NQO1-R	AAGAGGCTGCTTGGAGCAAA
ARID1A	ARID1A-F	TCATGCCTTCCAACCCAGAC
	ARID1A-R	GCTGGATACCTGCTGTTGCT
ACTB	ACTB-F	GACAGGATGCAGAAGGAGATTACT
	ACTB-R	TGATCCACATCTGCTGGAAGGT
